# Are you afraid of COVID‐19? Motivation and engagement in infection–prevention behaviour in a UK community cohort during the first 2 years of the COVID‐19 pandemic

**DOI:** 10.1111/bjhp.70034

**Published:** 2025-11-07

**Authors:** Rhiannon Phillips, Britt Hallingberg, Anna Torrens‐Burton, Fiona Wood, David Gillespie, Clare Glennan, Paul Sellars, Sherina Lowe, Aleysha Caffoor, Wouter Poortinga, Karin Wahl‐Jorgensen, Denitza Williams

**Affiliations:** ^1^ Cardiff School of Sport and Health Science Cardiff Metropolitan University Cardiff UK; ^2^ Division of Population Medicine, School of Medicine Cardiff University Cardiff UK; ^3^ Centre for Trials Research Cardiff University Cardiff UK; ^4^ School of Psychology Cardiff University Cardiff UK; ^5^ Welsh School of Architecture Cardiff University Cardiff UK; ^6^ Cardiff School of Journalism, Media and Culture Cardiff University Cardiff UK

**Keywords:** COM‐B, COVID‐19, infection–prevention behaviour, motivation, PRIME theory, risk perception, survey

## Abstract

**Objectives:**

To investigate the relationship between motivation and COVID‐19 infection–prevention behaviour during the first 2 years of the COVID‐19 pandemic.

**Design:**

Prospective longitudinal online survey in a UK community‐based cohort.

**Methods:**

During March/April 2020, *n* = 11,113 people, recruited via the HealthWise Wales research registry and social media advertising, completed the COVID‐19 Public Experiences (COPE) study baseline survey, with follow‐up at 3, 12, 18 and 24 months. Online questionnaires assessed COVID‐19 infection–prevention behaviour, perceived susceptibility, fear, personal control over infection transmission and reliability of information from media and government. Repeated‐measures ANOVA identified changes in motivation and behaviour over time. Multivariable regression models at each time point assessed associations between motivation and behaviour.

**Results:**

COVID‐19 infection–prevention behaviour and motivational variables (fear of COVID‐19, perceived susceptibility and perceived control) fluctuated over time as the disease and socio‐political environment changed, decreasing overall by 24 months. Regression models for association between motivational variables and COVID‐19 infection–prevention behaviour were statistically significant at three (*F*_(10, 5981)_ = 76.69, *p* < .001, adjusted *R* ^2^ .112), 12 (*F*_(11, 3732)_ = 48.40, *p* < .001, adjusted *R* ^2^ .122), 18 (*F*_(11, 3665)_ = 108.34, *p* < .001, adjusted *R* ^2^ .243) and 24 months (*F*_(11, 3355)_ = 136.20, *p* < .001, adjusted *R* ^2^ .306). Higher levels of fear, older age, lower perceived personal control over infection transmission, more trust in government and less trust in social media were associated with more infection–prevention behaviour.

**Conclusions:**

Motivation to engage in infection–prevention behaviour during a pandemic is multi‐factorial and dynamic. Beliefs about infection and trust in government and media need to be considered in developing effective communication strategies.

**Statement of contribution**

**What is already known on this subject?**

- Motivation is an important determinant of infection–prevention behaviour during pandemics
- Perceived risk of harm, effects of (in)action and self‐efficacy can influence motivation

**What does this study add?**

- Fear, perceived susceptibility and control were associated with COVID‐19‐prevention behaviour
- Motivation fluctuated as the COVID‐19 disease and socio‐political environment changed
- Motivation decreased overall by 24 months, but clinically vulnerable people remained fearful

## INTRODUCTION

The COVID‐19 pandemic led to high levels of mortality and morbidity, as well as economic and social disruption, with national lockdowns, border closures and pressure on healthcare services worldwide (McBride et al., 2021; McKibbin & Fernando, 2020; UK Health Security Agency, 2024; World Health Organization, 2020a, 2020b). Individual‐level behaviours recommended to reduce the spread of pandemic and epidemic disease included social distancing, avoiding touch and maintaining hygiene—particularly through hand washing (Michie et al., 2020). Human behaviour is especially important in the early stages of a pandemic where effective treatments and vaccinations are not yet available (Michie & West, 2020). Risk perception is an important motivational determinant of preventative and health‐promoting behaviour such as social distancing and hand washing during pandemics (Bish & Michie, 2010; Dryhurst et al., 2020; Wise et al., 2020; Yang & Cho, 2017). Risk perception is also multi‐dimensional and dynamic, varying between individuals and fluctuating over time as contexts change (e.g., infection rates, new variants, trust in government, mitigation policies; Phillips et al., 2022; Schneider et al., 2021; Tagini et al., 2021; Wang et al., 2021). Timing and context are important in understanding longitudinal shifts in risk perception during the COVID‐19 pandemic (Phillips et al., 2022; Savadori & Lauriola, 2022; Tagini et al., 2021; Wang et al., 2021). As such, longitudinal and holistic approaches are needed to understand how people perceive and respond to risk during pandemics to facilitate the planning of communication and public health strategies.

Perceptions of the risks and benefits of infection–prevention behaviour and perceptions of control over infection–prevention are important motivational factors for engagement in preventive action (Bish & Michie, 2010; Meng et al., 2023; Phillips et al., 2022). During a pandemic, affective risk appraisal can range from feelings of mild concern to intense fear (Tagini et al., 2021). Efficacy beliefs are an important component of motivation and can act jointly with risk perception to influence behaviour (Rimal & Real, 2003). Affective risk attitudes were found to be strongly associated with protective behaviour and remained consistently high during the epidemic and post‐epidemic phases of the COVID‐19 pandemic in an Italian study, while the overall decrease observed in perceived risk over time reflected a reduction in risk analysis (Savadori & Lauriola, 2022). Studies in the United Kingdom indicated that there was significant variation in risk perception corresponding to the easing and tightening of lockdown restrictions and following the introduction of widespread vaccination (Phillips et al., 2022; Schneider et al., 2021). Further, when considering the interaction between multiple risk reduction strategies, ‘risk compensation’ can occur, where perceived reduction in risk due to one action (e.g., wearing a face mask) may lead to a decrease in perceived need for other actions (e.g., maintaining physical distance; Luckman et al., 2021).

Both internal and external factors can influence motivation (West & Brown, 2013). Low trust in government, poor social trust and individualistic worldviews can reduce acceptance of pandemic mitigation strategies (Hanna et al., 2023; Siegrist & Bearth, 2021). However, these findings are not universal or linear; high trust in government can potentially decrease engagement in preventative behaviour due to an increased sense of security (Evensen et al., 2023; Liu et al., 2022). Exposure to media can influence motivation to engage in infection–prevention behaviour, depending on perceived accuracy of the information and trust in sources of information (Allington et al., 2021; Erfei et al., 2020; Oh et al., 2021; Schneider et al., 2021). Significant variability occurred in media coverage, trust in government and perceptions of engagement of others in infection–prevention behaviour over the course of the COVID‐19 pandemic (Evensen et al., 2023; Greenhawt et al., 2021; Liu & Yang, 2023; Shin & Youn, 2023; Zhou et al., 2023). The United Kingdom and its devolved nations (Wales, Northern Ireland and Scotland) were broadly similar in the types of public health intervention utilized during the COVID‐19 pandemic, but they diverged at various points in terms of the timing and detail of the deployment of interventions (British Medical Association, 2022b). A review of UK governments' public health response concluded that public health messaging and government communication were often incoherent and inconsistent, particularly in England (British Medical Association, 2022a). As such, it is vital that we adopt a holistic understanding of how motivation influences infection–prevention behaviour during the COVID‐19 pandemic, taking into consideration the dynamic and context‐sensitive nature of motivation.

### Objective

The purpose of this study was to investigate the association between motivation and infection–prevention behaviour over the first 2 years of the pandemic in a UK community cohort. We set out to investigate

1. How did motivation and behaviour vary over time during the COVID‐19 pandemic?
2. What is the relationship between motivation and self‐reported infection–prevention behaviour at different stages in the pandemic?
3. Are attitudes towards government and media associated with infection–prevention behaviour independently of infection‐related beliefs?

### Theoretical framework

The Capability, Opportunity and Motivation (COM‐B) (Michie et al., 2011) and the Plans, Responses, Impulses, Motives (wants and needs) and Evaluations (PRIME) theory of motivation (West & Brown, 2013) were used as a conceptual framework in this study to guide the selection of motivational variables and to facilitate interpretation of findings. The COM‐B is a widely used model of behaviour that can be used to understand the determinants of COVID‐19 infection–prevention behaviour (West et al., 2020). The model postulates that people's actions are influenced by their capability to enact a behaviour, a physical and social environment that provides opportunities that make that behaviour possible and motivation that energizes and directs behaviour. Motivation includes ‘automatic’ processes (wants, needs, desires, impulses and reflexes) and ‘reflective’ processes that involve self‐conscious planning and evaluation (e.g., beliefs about what is good or bad).

The PRIME theory of motivation provides further detail to the motivational component of COM‐B (West & Brown, 2013). PRIME theory proposes that the cause of behaviour is a balance between potentially competing impulses and inhibitions at a given moment. Habit, instinct and feelings of want or need control these processes. Evaluations are driven by judgement processes, wants, needs and plans. Motivation involves a strong perceived need to enact infection–prevention behaviour, and this must be sufficient to overcome competing wants or needs at that moment in time. Identity and modelling are important influences on behaviour, where people perceive enacting a behaviour to be valued by the social group(s) with which they identify and see other people with whom they identify and/or trust enacting the behaviour. To maintain infection–prevention behaviour in the longer term, rules and habits need to be developed (West et al., 2020).

## MATERIALS AND METHODS

Data from five online surveys conducted over a 24‐month period between March 2020 and April 2022 collected as part of the COVID‐19 Public Experiences (COPE) prospective longitudinal mixed methods study (Hallingberg et al., 2021; Phillips et al., 2021, 2022) was used in this analysis.

### Study population and recruitment

**FIGURE 1:**
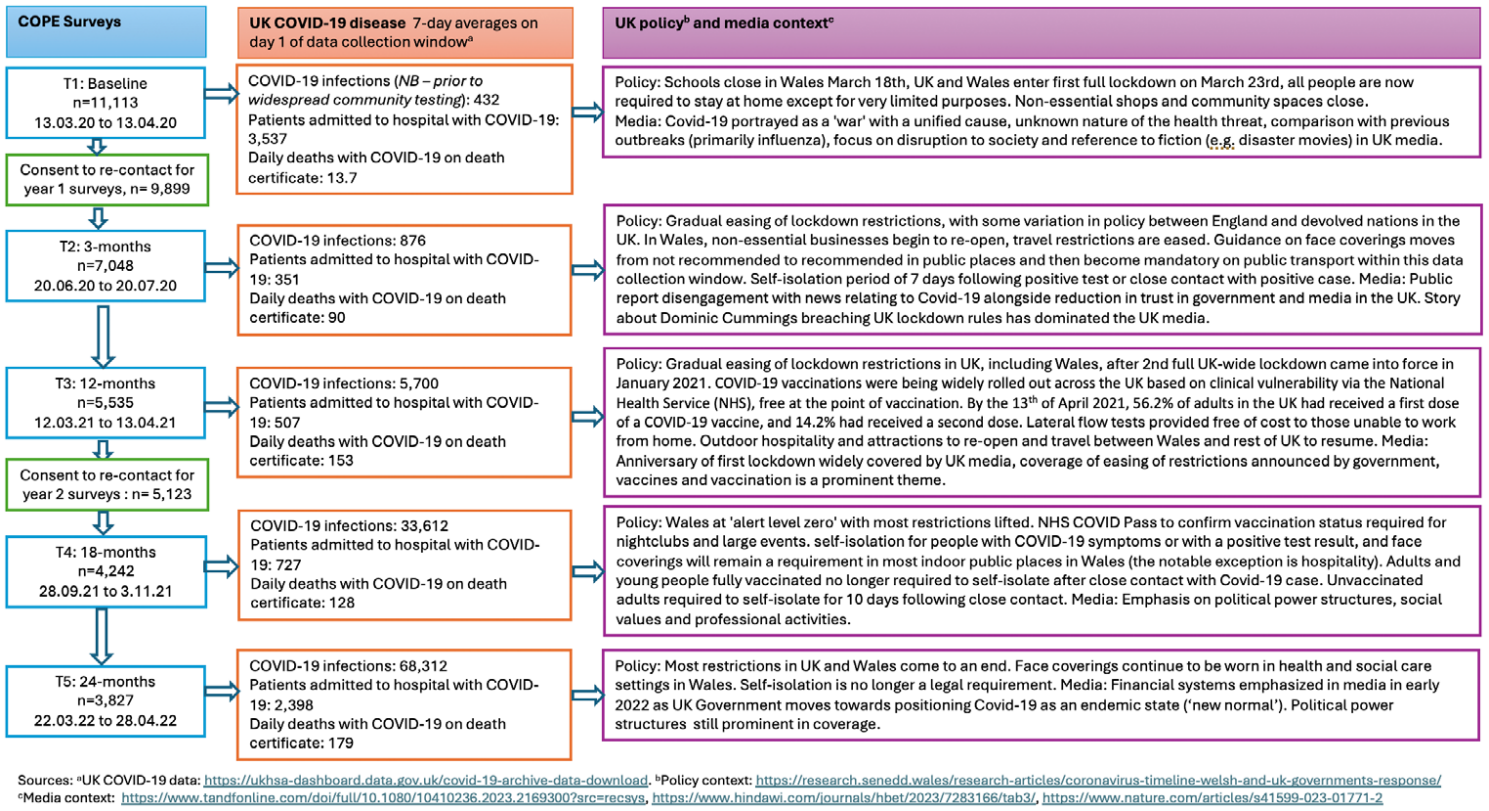
COPE surveys data flow and UK COVID‐19 disease, policy and media context during data collection windows.

The COPE cohort included 11,113 adults living in the United Kingdom at the time of enrolment, when the United Kingdom was entering its first national lockdown (13 March 2020–13 April 2020; Hallingberg et al., 2021; Phillips et al., 2021), recruited through social media adverts (Facebook®, Twitter® and Instagram®) and advertisement to the HealthWise Wales (HWW) research registry (Hurt et al., 2019). The majority of participants were recruited via HWW (78.5%, and therefore residing and/or receiving healthcare in Wales), female (69.2%), aged 50 and above (68%) and had a college degree or diploma (67%). As such, they were not representative of the general population in Wales or the United Kingdom (Phillips et al., 2021). Nonetheless, the cohort was well characterized, and in‐depth data were gathered from respondents on their actions, experiences, health and well‐being over a 2‐year period. Figure 1 provides a summary of survey data collection points and key infection, policy and media contextual considerations at each time point.

### Measures

The baseline survey included demographic questions on age, gender, highest level of education, ethnic group, children aged <18 living in the household and health‐related questions including pre‐existing medical conditions and seasonal flu vaccination uptake in the last 12 months. Participants were asked whether they had, or thought they may have had, COVID‐19, and whether this had been confirmed by a polymerase chain reaction or lateral flow test (if available). General psychological well‐being was assessed using the 4‐item version of the Patient Health Questionnaire (PHQ‐4) (Kroenke et al., 2009) at 3 and 12 months. Three items from the SF‐36 measure (Ware Jr. & Sherbourne, 1992) were included to assess psychological well‐being and general health.

*Perceived risk of COVID‐19* questions were adapted from studies during previous viral pandemics (Brug et al., 2004; Bults et al., 2011; de Zwart et al., 2007). Using 4‐point Likert scales, participants were asked to rate the perceived harmfulness of COVID‐19 in the next 12 months (not harmful at all to very harmful), how scared they were of COVID‐19 (not at all scared to very scared), how worried they were about COVID‐19 (not at all worried to very worried) and how likely they thought they were to get COVID‐19 in the next 12 months (very unlikely to very likely). We asked how often people thought about COVID‐19 on a 5‐point Likert scale (never to all the time) to assess attention to the COVID‐19 threat. The harmful, scared, worried and attention to COVID‐19 items were summed to provide a total ‘fear of COVID‐19’ score, with good internal reliability (Cronbach's alpha = .81; Phillips et al., 2022). Susceptibility was retained as a separate single‐item measure.

*Perceived behavioural control* is a concept that includes self‐efficacy (beliefs about ability to exercise control over events) and the controllability of an event or action (Ajzen, 2002). In this study, it was assessed using two items rated on a 5‐point scale (no control to complete control): perceived control over protecting themselves and members of their household from being infected by COVID‐19, and perceived control over helping to prevent the spread of COVID‐19 in their community. The two perceived control items were moderately correlated and were retained as separate items during analysis (*r* (7,006) = .36, *p* < .001; Phillips et al., 2022).

*COVID‐19 infection–prevention behaviour* was assessed at each time point from 3‐month follow‐up onwards using the COVID‐19 Infection‐Prevention Behaviour Scale developed as part of the COPE study (File S1). Participants were asked how often they had used each of eight COVID‐19 prevention behaviours in the last 2 weeks: staying home and only going out when essential, avoiding crowded indoor places (e.g., shops or offices); avoiding crowded outdoor spaces (e.g., popular parks or beauty spots), staying away from people who would be at higher risk from infection (e.g., older people, those with certain medical conditions), keeping at least 2 m (or about 6 feet) away from people who do not live in your household, avoiding close physical contact with others who do not live in your household (e.g., shaking hands, hugging, kissing), washing hands with soap and water when arriving home and avoiding touching your face (eyes, nose and mouth) with unwashed hands. Items were rated from 1 (never) to 5 (always). The items were summed to provide a total score, with a potential range of 8–40 and higher scores indicating more use of COVID‐19 infection–prevention behaviour. Internal reliability for the scale was good (Cronbach's alpha = .768).

*Trust in government and media* was assessed at 3‐, 12‐ and 18‐month follow‐up. Participants were asked to rate the reliability of COVID‐19 information from the UK Government, devolved national Government (where applicable), television, newspapers, radio and social media from 1 (not at all reliable) to 4 (very reliable). A mean ‘mainstream media’ score was calculated for the television, radio and newspaper items. Government and media variables were not included in the 24‐month survey, as it was a condensed survey to facilitate participant retention.

### Analysis

Statistical analysis was carried out using IBM® SPSS® Statistics version 27. Descriptive analysis was conducted to assess perceived risk and engagement with COVID‐19 infection–prevention behaviour at each time point. Repeated‐measures ANOVA models were produced for the perceived risk for all five timepoints, and for perceived behavioural control, COVID‐19 infection–prevention behaviour and perceived reliability of information from government and media scores for follow‐up surveys from 3 months onwards to assess changes in these variables over time.

Multivariable linear regression analysis was used to assess the independent association between self‐reported COVID‐19 infection–prevention behaviour and susceptibility, fear of COVID‐19 and perceptions of behavioural control over preventing infection from COVID‐19 and reducing the spread of COVID‐19 cross‐sectionally at each time point. Key individual characteristics and contextual variables were also entered into the models, including gender (male/female), level of education (college education/no college education), age category, self‐reported exposure to COVID‐19 since the previous survey, subjective general health and psychological distress (PHQ‐4) to account for factors that could influence perceived and/or actual risk of COVID‐19. Having received at least one COVID‐19 vaccination was added into the models at 12, 18 and 24 months. Missing data were excluded listwise. Based on Green's rule of thumb for multivariable regression, a sample of *N* > 50 + 8p, where p is the number of predictor variables, indicated that a minimum of *n* = 178 would be required for the planned analysis.

For the 3‐, 12‐ and 18‐month surveys, perceived reliability of information from UK government, devolved nation government, mainstream media (mean score for television, radio and newspapers) and social media was added to the regression models for people living in Wales to assess whether there was an additional direct influence of these contextual variables on infection–prevention behaviour. The government response to COVID‐19 in Wales operated on similar principles to England and the other devolved UK nations. However, there were some key differences in the timing and implementation of policies. Policymaking during the pandemic was shaped by political ideology, culture and demographic makeup and specific needs of the population, with the Welsh Government approach being more precautionary overall than that of the UK Government (Senedd Research, 2023). As the majority of participants were resident and/or receiving healthcare in Wales, this stage of the analysis was restricted to Welsh residents to enable us to examine the role of trust in devolved Welsh Government and central UK government.

## RESULTS

Demographic characteristics of the participants at each time point are provided in Table 1. At baseline, the majority of the 11,113 participants were female (69.2%), age 51 years or older (68.3%), white British (95.8%), had a pre‐existing medical condition (50.5%) and had received college (post‐18) education (67.1%). These demographic groups were over‐represented relative to the general population in Wales (File S2), and this pattern of over‐representation increased over the course of the study. Descriptive statistics for the risk perception and behavioural variables are provided in Table 2.

**TABLE 1:** Demographic characteristics of the COPE cohort at each survey time point.

Variable	Category	Baseline (*n* = 11,113)		3 months (*n* = 7048)		12 months (*n* = 5437)		18 months (*n* = 4242)		24 months (*n* = 3827)	
		*n*	%	*n*	%	*n*	%	*n*	%		
Gender	Female	7694	69.2	4781	67.8	3629	66.7	2774	65.4	2506	65.5
	Male	3359	30.2	2234	31.7	1786	32.8	1450	34.2	1310	34.2
	Missing/rather not say	60	.6	20	.3	22	.4	18	.4	11	.4
Age category	18–30	810	7.3	354	5	212	3.9	128	3	93	2.4
	31–40	1251	11.3	669	9.5	437	8	276	6.5	235	6.1
	41–50	1459	13.1	870	12.3	628	11.6	434	10.2	393	10.3
	51–60	2352	21.2	1483	21	1136	20.9	874	20.6	784	20.5
	61–70	3229	29.1	2244	31.8	1865	34.3	1566	36.9	1436	37.5
	71–80	1786	16.1	1277	18.1	1037	19.1	864	20.4	805	21
	81+	211	1.9	146	2.1	117	2.2	97	2.3	79	2.1
	Missing/rather not say	15	.2	5	.1	5	.1	3	.1	2	0
Ethnicity	White (Welsh, English, Scottish, Northern Irish, British)	10,514	94.6	6722	95.4	5174	95.2	4056	95.6	3667	95.8
	White other	316	2.8	185	2.6	160	2.9	117	2.8	103	2.7
	Black/African/Caribbean/Black British	14	.1	2	0	4	.1	2	0	1	0
	Asian/Asian British	58	.5	27	.4	16	.3	9	.2	10	.3
	Mixed/multiple ethnic groups	71	.6	42	.6	30	.6	19	.4	14	.4
	Other	22	.2	11	.2	9	.2	8	.2	6	.2
	Missing/Rather not say	118	1.1	59	.8	30	.6	31	.2	26	.6
Marital status	Single	1638	14.7	912	12.9	700	12.9	512	12.1	460	12
	Married or in civil partnership	6404	57.6	4249	60.3	3279	60.3	2576	60.7	2359	61.6
	Living with partner	1165	10.5	661	9.4	492	9	371	8.7	314	8.2
	Widowed, divorced or separated	1722	15.5	1136	16.1	893	16.4	726	17.1	643	16.8
	Other	92	.8	49	.7	36	.7	25	.6	21	.5
	Missing/rather not say	92	.8	41	.5	37	.7	32	.7	30	.8
Children under 18 years living in household	Yes	2161	19.4	1213	17.2	810	14.9	537	12.7	462	12.1
	No	8886	80	5798	82.3	4599	84.6	3684	86.8	3347	87.5
	Missing	66	.6	37	.5	28	.5	21	.5	18	.5
Highest level of education	No college (post 18) education	3418	30.7	2035	28.9	1540	28.3	1212	28.6	1094	28.5
	College (post 18) education	7458	67.1	4910	69.7	3769	69.3	2971	70	2689	70.2
	Missing	237	2.1	103	1.4	76	1.4	59	1.4	44	1.2
Employment status (participants were able to select all categories that apply)	Employed full time	3477	31.3	1987	28.2	1401	25.8	978	23.1	886	23.2
	Employed part time	2124	19.1	1317	18.7	995	18.3	756	17.8	641	16.7
	Unemployed	728	6.6	445	5.1	330	6	255	6	222	5.8
	Retired	4560	41.0	3256	46.2	2709	49.8	2272	53.6	2101	54.9
	In education or training	399	3.6	203	2.9	127	2.3	80	2.9	61	1.6
	Rather not say	116	1.0	50	.7	33	.6	20	.5	20	.5
Pre‐existing medical condition	Yes	5607	50.5	3623	51.4	2844	52.3	2238	52.8	2022	52.8
	No	5506	49.5	3425	48.6	2593	47.7	2004	47.2	1805	47.2
Flu vaccination in last 12 months	Yes	6089	54.8	4074	57.8	3220	59.2	2558	60.3	2339	61.1
	No	4968	44.7	2945	41.8	2195	40.4	1672	39.4	1479	38.6
	Missing	56	.5	29	.4	22	.4	12	.3	9	.2

**TABLE 2:** Descriptive statistics for motivational variables, trust in government and media and COVID‐19 infection–prevention behaviour at each time point (participants included in regression models).

	Baseline		3 months		12 months		18 months		24 months	
	Mean	SE	Mean	SE	Mean	SE	Mean	SE	Mean	SE
Perceived risk of COVID‐19										
Perceived susceptibility *(scored 0–3)*	1.59	.007	1.23	.008	.89	.009	1.17	.011	1.55	.012
Fear of COVID‐19 *(scored 0–13)*	9.01	.028	6.84	.034	6.734	.039	6.04	.043	5.16	.043
Perceived behavioural control										
Avoiding contracting COVID‐19 *(scored 0–4)*	N/A	N/A	2.29	.01	2.5	.011	2.24	.013	1.97	.015
Avoiding spreading COVID‐19 *(scored 0–4)*	N/A	N/A	2.1	.012	2.33	.013	2.07	.015	1.84	.017
Perceived reliability of government and media information										
Mainstream media	N/A	N/A	1.5	.008	1.6	.009	1.5	.012	N/A	N/A
Social media	N/A	N/A	.6	.009	.6	.011	.5	.011	N/A	N/A
UK government	N/A	N/A	1.9	.012	2.4	.011	2.2	.014	N/A	N/A
Devolved nation government (if applicable)	N/A	N/A	2.3	.010	2.5	.010	2.4	.012	N/A	N/A
Infection–prevention behaviour										
COVID‐19 infection prevention behaviour scale *(scored 8–40)*	N/A	N/A	35.1	.05	35.42	.062	29.33	.099	27.22	.115

*Note*: N/A indicates that this measure was not included at the relevant timepoint.

### Change in motivation and infection–prevention behaviour over time

Descriptive statistics and results of the repeated‐measures ANOVA for within‐person differences in risk perception and infection–prevention behaviour are shown in Table 3. The ANOVA models demonstrated a statistically significant within‐person effect of time for all variables of interest (*p* < .001, see File S3 for profile plots).

**TABLE 3:** Descriptive and repeated measures ANOVA models for motivational variables, trust in government and media and COVID‐19 infection–prevention behaviour for participants with data available at all time points.

Variable	Participants with data available at all survey time points	Baseline		3‐month follow‐up		12‐month follow‐up		18‐month follow‐up		24‐month follow‐up		Within‐person ANOVA	
	*n*	Mean	SE	Mean	SE	Mean	SE	Mean	SE	Mean	SE	*F*	*p*
Perceived risk of COVID‐19													
Perceived susceptibility (scored 0–3)	3091	1.51	.013	1.17	.012	.87	.011	1.16	.013	1.54	.013	678.66	<.001
Fear of COVID‐19 (scored 0–13)	3064	8.68	.052	6.71	.05	6.66	.051	5.99	.049	5.15	.048	1295.59	<.001
Perceived behavioural control (scored 0–4)													
Avoiding contracting COVID‐19	3160	N/A	N/A	2.35	.14	2.55	.14	2.26	.015	2	.016	375.39	<.001
Avoiding spreading COVID‐19	3127	N/A	N/A	2.14	.018	2.37	.018	2.11	.018	1.87	.018	186.54	<.001
Perceived reliability of information (scored 0–3)													
Mainstream media	3655	N/A	N/A	1.52	.11	1.66	.012	1.47	.012	N/A	N/A	153.20	<.001
Social media	2774	N/A	N/A	.57	.13	.57	.14	.50	.013	N/A	N/A	18.47	<.001
UK government	3470	N/A	N/A	1.88	.016	2.36	.014	2.17	.014	N/A	N/A	563.28	<.001
Devolved UK nation government	3408	N/A	N/A	2.27	.014	2.55	.012	2.46	.013	N/A	N/A	251.22	<.001
COVID‐19 infection prevention behaviour (scored 8–40)	3112	N/A	N/A	35.31	.08	35.61	.079	29.39	.011	27.24	.126	2168.71	<.001

*Note*: N/A indicates that this measure was not included at the relevant timepoint.

COVID‐19 infection–prevention behaviour increased slightly between the 3‐ and 12‐month surveys (mean difference = −.302, *p* < .001), decreased markedly between the 12‐ and 18‐month surveys (mean difference = 6.214, *p* < .001) and decreased further between the 18‐ and 24‐month surveys (mean difference = 2.150, *p* < .001).

Perceived susceptibility was high in this cohort at baseline when the UK had its first wave of COVID‐19 and entered its first national lockdown period. Perceptions of susceptibility then decreased at 3 and 12 months during periods when restrictions from the first and second national lockdowns were gradually being eased. Perceived susceptibility rose again at 18 months and again at 24 months, returning to the high levels observed at baseline. Fear of COVID‐19 scores decreased between baseline and three months but stayed at a high level at the three‐ and 12‐month surveys. Fear of COVID‐19 decreased again at 18 months, reaching its lowest level at 24 months.

Perceived control over protecting oneself from catching COVID‐19 and over the spread of COVID‐19 increased between the 3‐ and 12‐month surveys, coinciding with the widespread roll out of COVID‐19 vaccination in the United Kingdom. Perceived behavioural control then decreased by 18 months and fell further by 24‐month follow‐up, during a period in which restrictions were being eased, but community infection rates were increasing (Senedd Research, 2023; UK Government, 2022; UK Health Security Agency, 2024).

Mainstream media information was consistently perceived to be more reliable than social media (3 months mainstream media mean 1.5 vs. social media mean .6, SE .011, 95% CI .84–.88; 12 months mainstream media mean 1.6 vs. social media mean .6, SE .014, 95% CI 1.01–1.07; 18 months mainstream media mean 1.5 vs. social media mean .5, SE .016, 95% CI .94–1.00). Perceived reliability of mainstream media information was highest at the 12‐month survey (mean 1.6, SE .009), and lowest at 3 months (mean 1.5, SE .008) 18 months (mean 1.5, SE .012). Perceived reliability of social media was similar at three months (mean .6, SE .009) and 12 months (mean .6, SE .011) but reduced slightly at 18 months (mean .5, SE .011).

### Multivariable regression analysis

Multivariable linear regression models testing for association between motivational variables and COVID‐19 infection–prevention behaviour scores were statistically significant at 3 months (*F*_(10, 5981)_ = 76.69, *p* < .001, adjusted *R* ^2^ .112), 12 months (*F*_(11, 3732)_ = 48.40, *p* < .001, adjusted *R* ^2^ .122), 18 months (*F*_(11, 3665)_ = 108.34, *p* < .001, adjusted *R* ^2^ .243) and 24 months (*F*_(11, 3355)_ = 136.20, *p* < .001, adjusted *R* ^2^ .306). Beta, SE and 95% CIs for variables entered into the models are provided in Table 4.

**TABLE 4:** Cross‐sectional multivariable linear regression analysis for infection–prevention behaviour total scores at each time point.

Variable	3 months			12 months			18 months			24 months		
	*β*	SE	95% CI	*β*	SE	95% CI	*β*	SE	95% CI	*β*	SE	95% CI
Perceived risk												
Susceptibility	.401	.088	.**228, .573**	.452	.112	.**231, .672**	.257	.141	−.019, .533	−.148	.147	−.437, .141
Fear of COVID‐19	.486	.022	.**443, .529**	.457	.027	.**404, .509**	.939	.037	.**865, 1.012**	1.332	.045	**1.244, 1.419**
Perceived behavioural control												
Protecting self	.328	.076	.**179, .476**	.476	.097	.**286, .666**	.713	.125	.**467, .958**	.781	.134	.**519, 1.043**
Reducing spread	.333	.059	.**216, .450**	.289	.074	.**144, .433**	.588	.101	.**390, .786**	.563	.112	.**344, .782**
Health and well‐being												
Subjective general health	.183	.059	.**067, .299**	−.12	.072	−.261, .021	−.536	.098	**−.729, −.343**	−.549	.11	**−.765, −.334**
Psychological distress (PHQ‐4)	−.086	.022	**−.129, −.044**	−.121	.027	**−.174, −.068**	.025	.037	−.047, .097	.002	.042	−.081, .085
Believe they have had COVID‐19 in last 6 months	−.57	.291	−1.140, .000	−.558	.319	−1.184, .067	.83	.557	−.262, 1.922	1.059	.257	.**555, 1.562**
Received one or more COVID‐19 vaccinations	N/A	N/A	N/A	1.102	.234	.**644, 1.560**	3.36	.642	**2.210, 4.618**	2.543	.671	**1.227, 3.859**
Demographics												
Age category	.194	.046	.**105, .283**	.187	.066	.**057, .318**	.337	.08	.**179, .494**	.463	.09	.**287, .638**
College educated	.363	.138	.**093, .0633**	.259	.16	−.054, .572	.284	.225	−.157, .726	.11	.252	−.385, .605
Male/female	.829	.123	.**587, 1.072**	.589	.148	.**300, .879**	.878	.2	.**486, 1.270**	.417	.223	−.19, .854

*Note*: Statistically significant associations (*p* < .05) highlighted in bold, N/A indicates measure was not included at the relevant timepoint.

Fear of COVID‐19 and perceived behavioural control were consistently positively associated with COVID‐19 infection–prevention behaviour at all time points. From 12 months onwards, when widespread vaccine roll‐out was underway in the United Kingdom, having received a COVID‐19 vaccination was positively associated with infection–prevention behaviour. Self‐reported COVID‐19 infection in the last 6 months was associated with more infection–prevention behaviour at 24 months.

Psychological distress was associated with less infection–prevention behaviour at 3 and 12 months. Better general health was associated with more infection–prevention behaviour at 3 months. The direction of this association was reversed at 18‐ and 24‐month follow‐up, with poorer general health being associated with more infection–prevention behaviour. Increasing age was associated with more COVID‐19 prevention behaviour at all time points. Being female was associated with more infection–prevention behaviour at 3, 12 and 18 months, but not at 24 months. College education was associated with more infection–prevention behaviour at baseline, but not at follow‐up.

Regression models focusing on participants who were resident and receiving healthcare in Wales were carried out to investigate the effect of including the perceived reliability of information from media and government variables. The models were statistically significant at 3 (*F*_(14, 4697)_ = 46.34, *p* < .001, adjusted *R* ^2^ .119), 12 (*F*_(15, 2451)_ = 29.241, *p* < .001, adjusted *R* ^2^ .147) and 18 months (*F*_(15, 2582)_ = 58.371, *p* < .001, adjusted *R* ^2^ .249). Beliefs that social media information was reliable were associated with decreased reports of infection–prevention behaviour at all three time points. There was no association between perceived reliability of mainstream media and infection prevention behaviour. Perceived reliability of information from Welsh Government was positively associated with infection–prevention behaviour at all timepoints, whereas the reliability of UK government information was only associated with infection–prevention behaviour at the 12‐month time point (following an extended UK‐wide period of lockdown at the beginning of 2021). The inclusion of the government and media reliability variables did not alter the associations observed in the original models reported above (File S4).

## DISCUSSION

In line with COM‐B and PRIME Theory our findings indicated that in the COPE study cohort motivation to engage in COVID‐19 infection–prevention behaviour was multi‐factorial, with important differences observed between individuals and over time as the physical and social environment shifted. Fear of COVID‐19, perceptions of personal control over COVID‐19 transmission, and age were consistently associated with infection–prevention behaviour during all follow‐up surveys. Fear, perceived control and infection–prevention behaviour fluctuated as the external environment changed but decreased overall between baseline and 24‐month follow‐up. The amount of variance in infection–prevention behaviour that was explained by the motivation‐based regression models increased from 11.2% at baseline to 30.6% at 24 months, coinciding with a shift in responsibility for preventing the spread of infection from the state to the individual as lockdowns were lifted and public health protection measures removed (Hargreaves & Logie, 2020). Less infection–prevention behaviour was reported by those who perceived social media sources to be more reliable, whereas more infection–prevention behaviour was reported by those who perceived information from Welsh Government to be reliable, indicating that trust as well as infection‐related beliefs were important in understanding COVID‐19 infection–prevention behaviour. These findings highlight the need to consider the multi‐dimensional and dynamic nature of motivation when planning public health interventions and communication strategies during a pandemic.

As the COVID‐19 pandemic progressed there were dramatic changes in SARS‐CoV‐2 (the virus causing COVID‐19 disease) prevalence and emergence of new variants, COVID‐19‐related morbidity and mortality, availability of treatments, testing and vaccination, and the implementation of public health interventions to prevent transmission including a series of national and local lockdowns (Senedd Research, 2023; World Health Organization, 2020a). In the present study, while fear of COVID‐19, perceptions of personal control and engagement with infection–prevention behaviour had decreased by 24‐month follow‐up overall, perceived susceptibility was relatively high. COVID‐19 infection rates, hospitalization and deaths were high in the United Kingdom during the 24‐month survey (UK Government, 2022; UK Health Security Agency, 2024), indicating a discrepancy between motivation and objective risk of COVID‐19‐related harm at this point in time.

Interventions to prevent COVID‐19 were costly and effortful for individuals and societies, often with a disproportionate impact on socio‐economically deprived communities and people at risk of mental health problems and social isolation (UK Health Security Agency, 2024; Wong et al., 2020; World Health Organization, 2020b; Wright et al., 2020). A major challenge in the context of COVID‐19 is to assess what an ‘appropriate’ level of perceived risk and engagement in infection–prevention behaviour would be at different points in time, considering the external context and individual characteristics and circumstances. Male sex, older age, ethnic minority groups, co‐morbidity, obesity and smoking are associated with increased mortality from COVID‐19 (Aldridge et al., 2020; Jordan et al., 2020; Tazerji et al., 2022). Female gender, increasing age, co‐morbidity, high BMI, smoking and previous hospital/intensive care admission are associated with increased risk of developing post‐COVID condition or ‘long‐COVID’ (Tsampasian et al., 2023). Those who continued to engage in infection–prevention behaviour at the 2‐year follow‐up point in the current study were more likely to be at higher risk from harm, including older people and those with poorer general health. Infection–prevention behaviour comes at a personal and financial cost to the individual and to society and can be difficult to sustain (Hampton et al., 2020). Infection–prevention habits may need to be sustained or changed depending on the ongoing risk on a population level and to particular individuals. Public health responses to pandemics need to include an effective exit strategy that takes into consideration the well‐being of those who are at high risk of infection‐related harm and of people who have developed ingrained beliefs and habits that could make re‐integration into their communities and daily activities more challenging.

Vaccination against COVID‐19 is associated with a reduction in SARS‐CoV‐2 infection, mortality, reduced disease severity and reduced risk of post‐COVID conditions (Malden et al., 2024). Perceptions of recent natural exposure to COVID‐19 were not associated with infection–prevention behaviour during the early stages of the pandemic, but at 24 months having had COVID‐19 in the last 6 months was associated with more infection–prevention behaviour. The reasons for this are unclear, but it is possible that contracting the illness despite having been vaccinated and/or having previously been infected may have increased respondents' sense of vulnerability. Previous research has demonstrated a lack of a consistent effect of personal experience of COVID‐19, where prior infection can reduce (Smith et al., 2020) and increase (Schneider et al., 2021) perception of risk. Following the widespread rollout of COVID‐19 vaccines in the United Kingdom (from the 12‐month COPE survey onwards), having been vaccinated was associated with more infection–prevention behaviour. As such, our findings did not suggest a risk compensation effect following either natural exposure or vaccination against COVID‐19 had occurred in this cohort.

The perceived reliability of information from the Welsh Government was positively associated with infection–prevention behaviour at all timepoints, whereas the reliability of the UK government was only independently associated with infection–prevention at the 12‐month data collection point. This survey took place as restrictions from a prolonged UK‐wide lockdown in the first quarter of 2021 were being gradually eased, and as such UK Government policy had a considerable impact on people living in Wales. At other points during the pandemic, the devolved nation governments in the United Kingdom had more control and flexibility in the implementation of COVID‐19‐related policies and organization of key services and infrastructure (Paun et al., 2020; Senedd Research, 2023).

Perceived reliability of mainstream media was not associated with infection–prevention behaviour in this study. However, perceptions that social media information was highly reliable were associated with less infection–prevention behaviour. The spread of misinformation and conspiracy beliefs via social media has been noted as problematic during the pandemic (Allington et al., 2021). Coupled with distrust of government, high trust in social media information can have a significant impact on engagement with infection–prevention behaviour independently of an individual's infection‐related beliefs. This highlights the need to build trust in government and official channels of information during pandemics.

### Strengths and limitations

This large longitudinal prospective study provides novel insights into how the relationship between motivation and COVID‐19 infection–prevention behaviour changed over a 2‐year period. The COPE cohort had a high proportion of older adults and people with long‐term conditions relative to the Welsh and UK general population (Phillips et al., 2021), and as such were a relatively high‐risk population for potential harm from COVID‐19. There were insufficient numbers of people from ethnic minority communities in this cohort to enable meaningful analysis by ethnicity (Phillips et al., 2021). Analysis of change in behaviour and motivation over time focused on people who had completed the surveys at all time points only, introducing further sampling bias. As such, it is not possible to generalize our findings to the general population in Wales or the United Kingdom. Standardized methods for assessing COVID‐19 risk perception and prevention behaviour were not available at the outset of the study. Initial validation indicated that the measures developed for this study were appropriate for the purposes of this research. Future pandemic research needs to adopt a more unified approach to high‐quality measurement of infection–prevention behaviour and motivational variables to enable more consistency, comparison and opportunity for collaboration between studies and across geographical areas.

## CONCLUSIONS

Motivation to engage in COVID‐19 prevention behaviour is multi‐factorial and dynamic, requiring an understanding of both automatic and reflective processes. Pandemic response planning needs to consider dynamic individual and contextual influences on motivation. Beliefs about infection‐related harm, perceptions of personal control over infection–prevention, and trust in government and social media need to be considered in developing effective communication strategies. Particular attention needs to be given to robust pandemic exit planning, with consideration of infection–prevention habits that may need to be sustained or altered and the well‐being of those at higher risk of infection‐related harm when population‐level health protection interventions are reduced/removed.

## AUTHOR CONTRIBUTIONS

**Rhiannon Phillips:** Conceptualization; investigation; funding acquisition; writing – original draft; methodology; formal analysis; project administration; supervision; data curation. **Britt Hallingberg:** Conceptualization; investigation; funding acquisition; writing – review and editing; methodology; project administration. **Anna Torrens‐Burton:** Conceptualization; investigation; writing – review and editing; methodology; data curation. **Fiona Wood:** Conceptualization; investigation; funding acquisition; writing – review and editing. **David Gillespie:** Conceptualization; methodology; investigation; funding acquisition; writing – review and editing. **Clare Glennan:** Conceptualization; investigation; writing – review and editing; methodology. **Paul Sellars:** Conceptualization; investigation; writing – review and editing. **Sherina Lowe:** Conceptualization; investigation; writing – review and editing; project administration. **Aleysha Caffoor:** Conceptualization; investigation; writing – review and editing; project administration. **Wouter Poortinga:** Conceptualization; investigation; funding acquisition; writing – review and editing; methodology. **Karin Wahl‐Jorgensen:** Conceptualization; investigation; funding acquisition; writing – review and editing; methodology. **Denitza Williams:** Conceptualization; investigation; funding acquisition; writing – review and editing; methodology; project administration; supervision.

## FUNDING INFORMATION

Phases 1 and 2 of this research were supported by internal resources at Cardiff Metropolitan University, Cardiff University, HealthWise Wales and PRIME Centre Wales. This included allowing core team members time to design, set up and conduct the baseline and 3‐month data collection. In August 2020, we were awarded a Sêr Cymru III Tackling COVID‐19 grant (Project number WG 90) to cover the period between the 1 August 2020 and 30 April 2021 to support our Phase 3 follow‐up data collection, analysis and dissemination.

## Supporting information


File S1.

File S2.

File S3.

File S4.

## Data Availability

Individual‐level data from our COPE online survey and qualitative data will not be made publicly available due to data security and ethical considerations. The data provided are of a detailed and sensitive nature. Our public contributors expressed concerns about privacy and security during the development and recruitment stages of this research and did not feel that it was appropriate for individual‐level data to be made publicly available. Anonymized data from the COPE study can be made available by the authors on reasonable request, subject to approval from the COPE Study Management Group and Cardiff Metropolitan University Applied Psychology Ethics Panel.
