# Supplementary material for: Are you afraid of COVID‐19? Motivation and engagement in infection–prevention behaviour in a UK community cohort during the first 2 years of the COVID‐19 pandemic
Source: Br J Health Psychol. 2025 Nov 7;30(4):e70034. doi: 10.1111/bjhp.70034 (PMC12593319; doi:10.1111/bjhp.70034)
Supplement: Supplementary file 1 — File S1. [file BJHP-30-0-s002.docx]

# Supplementary File 1: Principal Components Analysis for the COVID-19 Infection Prevention Behaviour Scale

The items for inclusion in the Covid-19 Infection-Prevention Behaviour Scale were developed using pilot items and free-text data collected during the baseline COPE survey. Government guidance and legislation changed very rapidly during the COPE baseline data collection period in March/April 2020, meaning that some items rapidly became irrelevant, and others needed to be incorporated into future surveys. Some of the original items were also only applicable to a sub-set of the cohort, such as items relating to working from home, keeping children home from school/nursery, avoiding public transport (many did not use public transport or were staying at home/self-isolating), and washing hands while outside the home (not applicable to those staying at home and/or self-isolating). Additional ad-hoc items were added to follow-up surveys as availability of PCR, lateral flow tests, and vaccination changed.

Ten items considered for inclusion in the COVID-19 Infection Prevention Behaviour Scale based on applicability to the whole cohort (e.g. removing items not only applicable to working people or those with children living in the household) and sufficient variation to be evident in a rapidly-changing context of easing and tightening of lockdown restrictions. Exploratory Principle Components Analysis was conducted using the three-month follow-up data (n=6,038 cases). Orthogonal (Varimax) rotation was applied but this did not improve interpretation of the matrix, and as such the unrotated matrix was retained.

Principal Components Analysis indicated that the eight items detailed below loaded onto one component, with good internal reliability (Cronbach’s alpha = 0.768). Two further items, using hand sanitiser and wearing a face covering or mask when outside the home, loaded onto a second component but internal reliability was below acceptable levels (Cronbach’s alpha = 0.402). These actions were also subject to changing guidance and availability during the pandemic, and as such these were not included in the current analysis.

**Factor Analysis of COVID-19 infection prevention behaviour items**

| **KMO and Bartlett's Test** | | |
| --- | --- | --- |
| Kaiser-Meyer-Olkin Measure of Sampling Adequacy. | | .820 |
| Bartlett's Test of Sphericity | Approx. Chi-Square | 11691.615 |
|  | df | 45 |
|  | Sig. | <.001 |

| **Communalities** | | |
| --- | --- | --- |
|  | Initial | Extraction |
| 3m How often: Staying home? | 1.000 | .492 |
| 3m How often: Avoiding crowded indoor places? | 1.000 | .584 |
| 3m How often: Avoiding crowded outdoor spaces? | 1.000 | .541 |
| 3m How often: Staying away from people who might be higher risk? | 1.000 | .350 |
| 3m How often: Keeping at least 2 metres away? | 1.000 | .471 |
| 3m How often: Avoiding close physical contact? | 1.000 | .318 |
| 3m How often: Washing hands with soap and water when coming home? | 1.000 | .478 |
| 3m How often: Avoiding touching face? | 1.000 | .591 |
| 3m How often: Using hand sanitiser? | 1.000 | .591 |
| 3m How often: Using facemask? | 1.000 | .262 |
| Extraction Method: Principal Component Analysis. | | |

| **Total Variance Explained** | | | | | | | |
| --- | --- | --- | --- | --- | --- | --- | --- |
| Component | Initial Eigenvalues | | | Extraction Sums of Squared Loadings | | | Rotation Sums of Squared Loadings |
|  | Total | % of Variance | Cumulative % | Total | % of Variance | Cumulative % | Total |
| 1 | 3.296 | 32.964 | 32.964 | 3.296 | 32.964 | 32.964 | 2.755 |
| 2 | 1.381 | 13.815 | 46.779 | 1.381 | 13.815 | 46.779 | 1.923 |
| 3 | .996 | 9.956 | 56.735 |  |  |  |  |
| 4 | .849 | 8.491 | 65.226 |  |  |  |  |
| 5 | .741 | 7.407 | 72.633 |  |  |  |  |
| 6 | .665 | 6.654 | 79.287 |  |  |  |  |
| 7 | .594 | 5.937 | 85.224 |  |  |  |  |
| 8 | .530 | 5.297 | 90.521 |  |  |  |  |
| 9 | .508 | 5.080 | 95.600 |  |  |  |  |
| 10 | .440 | 4.400 | 100.000 |  |  |  |  |

| **Total Variance Explained** | | |
| --- | --- | --- |
| Component | Rotation Sums of Squared Loadings | |
|  | % of Variance | Cumulative % |
| 1 | 27.551 | 27.551 |
| 2 | 19.228 | 46.779 |

| Extraction Method: Principal Component Analysis. |
| --- |

| **Component Matrix**^a^ | | |
| --- | --- | --- |
|  | Component | |
|  | 1 | 2 |
| 3m How often: Avoiding crowded indoor places? | .691 | -.327 |
| 3m How often: Keeping at least 2 metres away? | .678 | -.108 |
| 3m How often: Avoiding crowded outdoor spaces? | .662 | -.322 |
| 3m How often: Staying home? | .639 | -.288 |
| 3m How often: Avoiding touching face? | .589 | .494 |
| 3m How often: Staying away from people who might be higher risk? | .552 | -.214 |
| 3m How often: Washing hands with soap and water when coming home? | .550 | .419 |
| 3m How often: Avoiding close physical contact? | .543 | -.154 |
| 3m How often: Using facemask? | .367 | .357 |
| 3m How often: Using hand sanitiser? | .363 | .678 |
| Extraction Method: Principal Component Analysis.^a^ | | |
| a. 2 components extracted. | | |

| **Rotated Component Matrix**^a^ | | |
| --- | --- | --- |
|  | Component | |
|  | 1 | 2 |
| 3m How often: Avoiding crowded indoor places? | .759 | .091 |
| 3m How often: Avoiding crowded outdoor spaces? | .731 | .079 |
| 3m How often: Staying home? | .695 | .096 |
| 3m How often: Keeping at least 2 metres away? | .631 | .269 |
| 3m How often: Staying away from people who might be higher risk? | .581 | .112 |
| 3m How often: Avoiding close physical contact? | .541 | .158 |
| 3m How often: Using hand sanitiser? | -.053 | .767 |
| 3m How often: Avoiding touching face? | .236 | .732 |
| 3m How often: Washing hands with soap and water when coming home? | .243 | .647 |
| 3m How often: Using facemask? | .121 | .497 |
| Extraction Method: Principal Component Analysis. Rotation Method: Varimax with Kaiser Normalization. ^a^ | | |
| a. Rotation converged in 3 iterations. | | |

| **Component Transformation Matrix** | | |
| --- | --- | --- |
| Component | 1 | 2 |
| 1 | .847 | .532 |
| 2 | -.532 | .847 |
| Extraction Method: Principal Component Analysis. Rotation Method: Varimax with Kaiser Normalization. | | |

**Reliability analysis for COVID-19 infection-prevention scale items: Component 1**

| **Case Processing Summary** | | | |
| --- | --- | --- | --- |
|  | | N | % |
| Cases | Valid | 6038 | 54.3 |
|  | Excluded^a^ | 5075 | 45.7 |
|  | Total | 11113 | 100.0 |
| a. Listwise deletion based on all variables in the procedure. | | | |

| **Reliability Statistics** | |
| --- | --- |
| Cronbach's Alpha | N of Items |
| .768 | 8 |

| **Item Statistics** | | | |
| --- | --- | --- | --- |
|  | Mean | Std. Deviation | N |
| 3m How often: Staying home? | 4.34 | .878 | 6038 |
| 3m How often: Avoiding crowded indoor places? | 4.40 | .799 | 6038 |
| 3m How often: Avoiding crowded outdoor spaces? | 4.46 | .896 | 6038 |
| 3m How often: Staying away from people who might be higher risk? | 4.49 | .887 | 6038 |
| 3m How often: Keeping at least 2 metres away? | 4.62 | .627 | 6038 |
| 3m How often: Avoiding close physical contact? | 4.83 | .532 | 6038 |
| 3m How often: Washing hands with soap and water when coming home? | 4.75 | .587 | 6038 |
| 3m How often: Avoiding touching face? | 3.97 | .947 | 6038 |

| **Item-Total Statistics** | | | | |
| --- | --- | --- | --- | --- |
|  | Scale Mean if Item Deleted | Scale Variance if Item Deleted | Corrected Item-Total Correlation | Cronbach's Alpha if Item Deleted |
| 3m How often: Staying home? | 31.53 | 11.165 | .517 | .734 |
| 3m How often: Avoiding crowded indoor places? | 31.46 | 11.230 | .579 | .722 |
| 3m How often: Avoiding crowded outdoor spaces? | 31.40 | 10.967 | .540 | .729 |
| 3m How often: Staying away from people who might be higher risk? | 31.38 | 11.589 | .430 | .751 |
| 3m How often: Keeping at least 2 metres away? | 31.24 | 12.224 | .537 | .735 |
| 3m How often: Avoiding close physical contact? | 31.03 | 13.109 | .410 | .754 |
| 3m How often: Washing hands with soap and water when coming home? | 31.11 | 12.934 | .401 | .754 |
| 3m How often: Avoiding touching face? | 31.89 | 11.530 | .396 | .760 |

**Reliability analysis for COVID-19 infection-prevention scale items: Component 2**

NB: This component was not used in the analysis described in this manuscript due to low reliability.

| **Case Processing Summary** | | | |
| --- | --- | --- | --- |
|  | | N | % |
| Cases | Valid | 6619 | 59.6 |
|  | Excluded^a^ | 4494 | 40.4 |
|  | Total | 11113 | 100.0 |
| a. Listwise deletion based on all variables in the procedure. | | | |

| **Reliability Statistics** | |
| --- | --- |
| Cronbach's Alpha | N of Items |
| .408 | 2 |

| **Item Statistics** | | | |
| --- | --- | --- | --- |
|  | Mean | Std. Deviation | N |
| 3m How often: Using hand sanitiser? | 3.80 | 1.132 | 6619 |
| 3m How often: Using facemask? | 2.49 | 1.410 | 6619 |

| **Item-Total Statistics** | | | | |
| --- | --- | --- | --- | --- |
|  | Scale Mean if Item Deleted | Scale Variance if Item Deleted | Corrected Item-Total Correlation | Cronbach's Alpha if Item Deleted |
| 3m How often: Using hand sanitiser? | 2.49 | 1.988 | .262 | . |
| 3m How often: Using facemask? | 3.80 | 1.281 | .262 | . |
