# Supplementary material for: Are you afraid of COVID‐19? Motivation and engagement in infection–prevention behaviour in a UK community cohort during the first 2 years of the COVID‐19 pandemic
Source: Br J Health Psychol. 2025 Nov 7;30(4):e70034. doi: 10.1111/bjhp.70034 (PMC12593319; doi:10.1111/bjhp.70034)
Supplement: Supplementary file 2 — File S2. [file BJHP-30-0-s003.docx]

# Supplementary File 2: Table of COPE demographic profile compared with Welsh and UK general population statistics

| **Characteristic** | **Category** | **COPE cohort** (n=11,113) | **Wales**  (Population aged 16+ = 2,589,044) |
| --- | --- | --- | --- |
|  |  | **%** | **%** |
| Sex (StatsWales, 2019) | Male | 31 | 49 |
|  | Female | 69 | 51 |
| Age group (StatsWales, 2019)* | 61+ years, COPE cohort  65+, Welsh and UK population data | 47 | 26 |
| Ethnicity (Office of National Statistics, 2020; StatsWales, 2019) | White | 98 | 96 |
|  | Other | 2 | 4 |
| Marital status (Office for National Statistics, 2020b) | Married or civil partnered | 58 | N/A |
| Highest level of education (Office for National Statistics, 2017) | People who have achieved a higher education qualification | 67 | 47 |
| Flu vaccination in the last 12 months (Public Health England, 2020; Public Health Wales, 2020)* | Adults <60 years with a pre-existing condition who had received a flu vaccination | 25 | 44 |
|  | Older adults who had received a flu vaccination (COPE aged 60+, UK and Welsh population aged 65+) | 62 | 69 |
| Pre-existing medical conditions (Office for National Statistics, 2020a; StatsWales, 2020) | Proportion reporting any longstanding health condition(s). | 51 | 48 |
| General health – self-reported (NHS Digital, 2019; StatsWales, 2020) | COPE - good, very good, or excellent  Population data – good or very good | 81 | 71 |
|  | COPE – poor  Population data - bad or very bad | 5 | 9 |

**COPE age bands and general health self-evaluation categories do not correspond directly to publicly available population data and closest approximation of categories has been provided.*

**Sources:**

NHS Digital. (2019). *National Survey for Enlgand, 2019.* <https://files.digital.nhs.uk/23/6B5DEA/HSE19-Adult-health-rep.pdf>

Office for National Statistics. (2017). *Graduates in the UK labour market*. <https://www.ons.gov.uk/employmentandlabourmarket/peopleinwork/employmentandemployeetypes/articles/graduatesintheuklabourmarket/2017#graduates-across-areas-of-great-britain>

Office for National Statistics. (2020a). *People with long-term health conditions, UK: January to December 2019*. <https://www.ons.gov.uk/peoplepopulationandcommunity/healthandsocialcare/conditionsanddiseases/adhocs/11478peoplewithlongtermhealthconditionsukjanuarytodecember2019>

Office for National Statistics. (2020b). *Population estimates by marital status and living arrangements, England and Wales: 2019*. <https://www.ons.gov.uk/peoplepopulationandcommunity/populationandmigration/populationestimates/bulletins/populationestimatesbymaritalstatusandlivingarrangements/2019#marital-status-and-living-arrangements-data>

Office of National Statistics. (2020). *Research report on population estimates by ethnic group and religion (Census 2011 data)*. <https://www.ons.gov.uk/peoplepopulationandcommunity/populationandmigration/populationestimates/articles/researchreportonpopulationestimatesbyethnicgroupandreligion/2019-12-04>

Public Health England. (2020). *Seasonal influenza vaccine uptake in GP patients: winter season 2019 to 2020*. <https://assets.publishing.service.gov.uk/government/uploads/system/uploads/attachment_data/file/912099/Annual-Report_SeasonalFlu-Vaccine_GPs_2019-20_FINAL_amended.pdf>

Public Health Wales. (2020). *Seasonal influenza in Wales 2019/20 Annual Report*. <https://www.wales.nhs.uk/sites3/page.cfm?orgid=457&pid=55714>

StatsWales. (2019). *Population and migration estimates* <https://statswales.gov.wales/Catalogue/Population-and-Migration/Population/Estimates>

StatsWales. (2020). *National Adult Survey for Wales, Sept 2019 - March 2020*. <https://statswales.gov.wales/Catalogue/National-Survey-for-Wales/Population-Health/Adult-general-health-and-illness>
