# Supplementary material for: Are you afraid of COVID‐19? Motivation and engagement in infection–prevention behaviour in a UK community cohort during the first 2 years of the COVID‐19 pandemic
Source: Br J Health Psychol. 2025 Nov 7;30(4):e70034. doi: 10.1111/bjhp.70034 (PMC12593319; doi:10.1111/bjhp.70034)

# Supplementary File 3: Means plots for within-person contrasts by time in ANOVA

**Figure S1:** COVID-19 infection-prevention behaviour scores

**
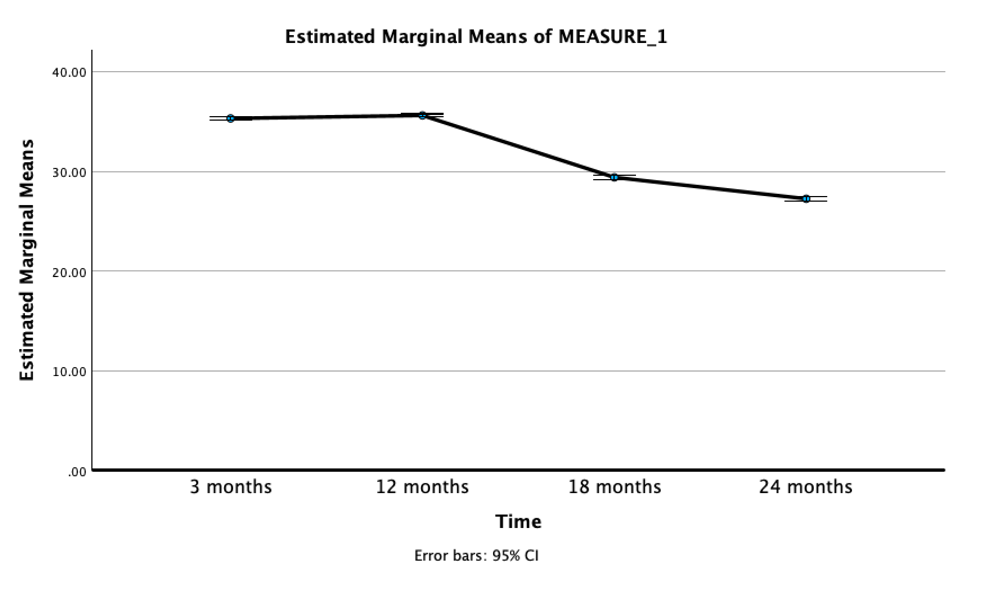
**

**Figure S2:** Perceived Susceptibility to COVID-19

**
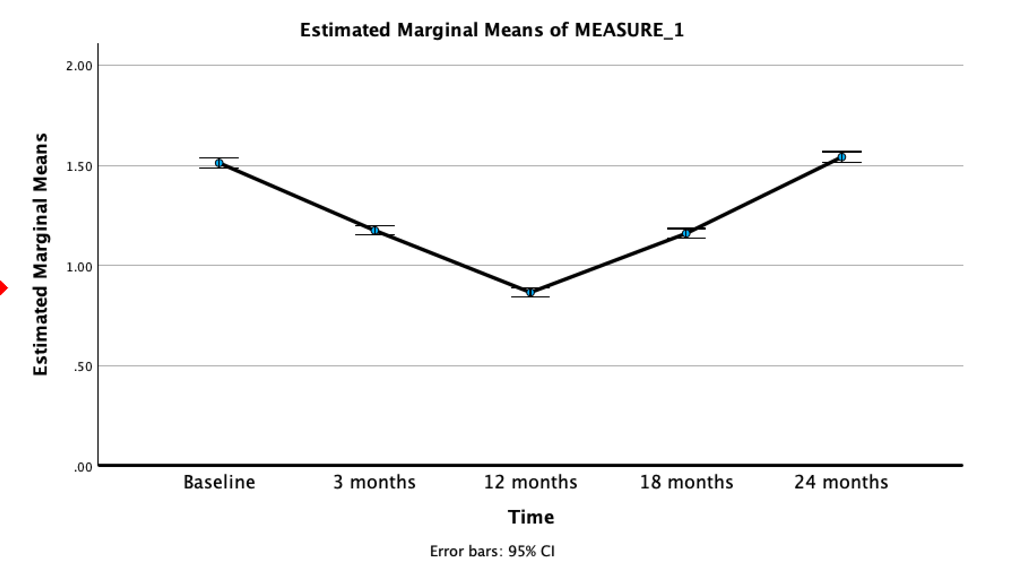
**

**Figure S3:** Fear of COVID-19

**
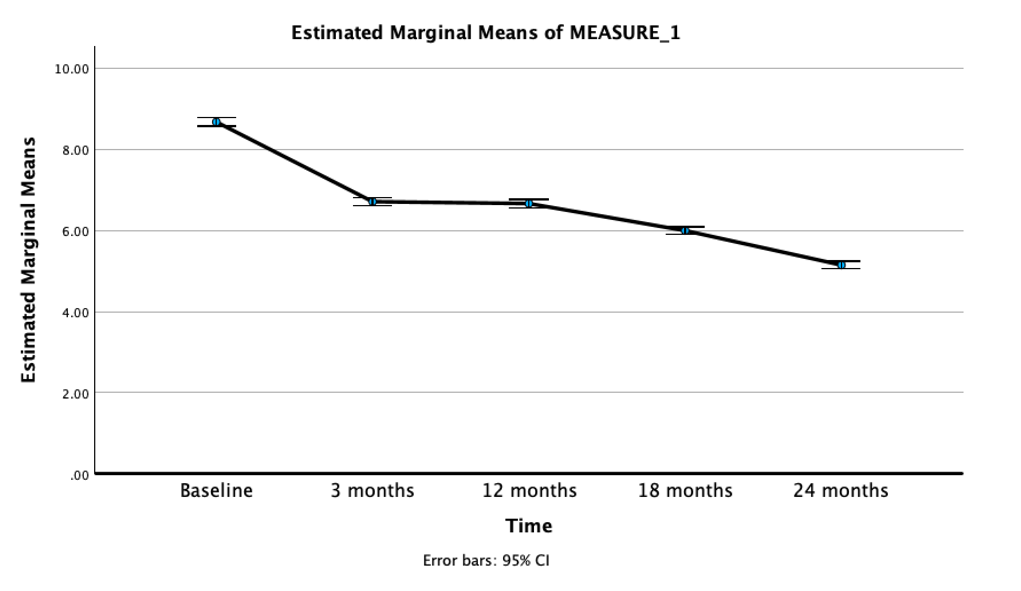
**

**Figure S4:** Perceived behavioural control: preventing self and family from getting COVID-19

**
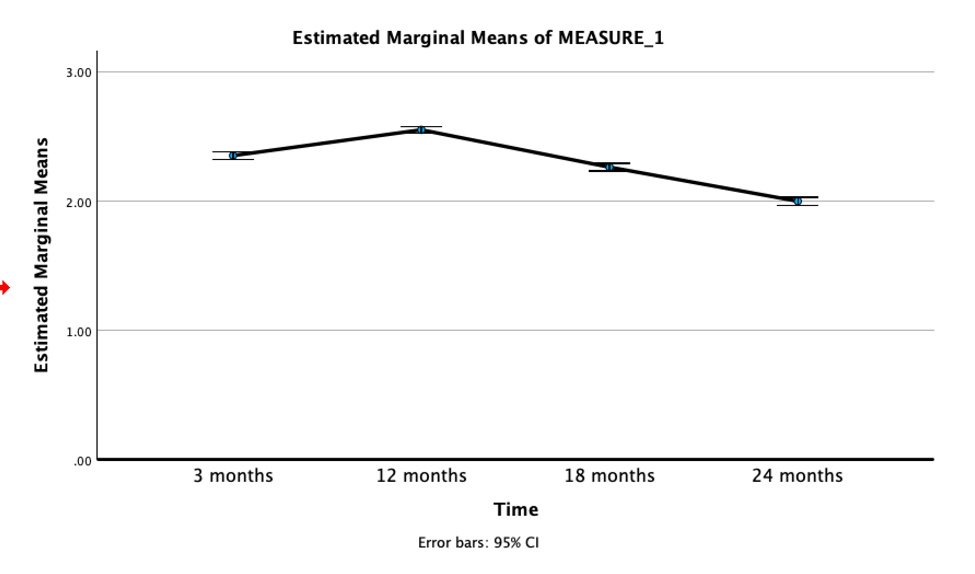
**

**Figure S5:** Perceived behavioural control: preventing the spread of COVID-19

**
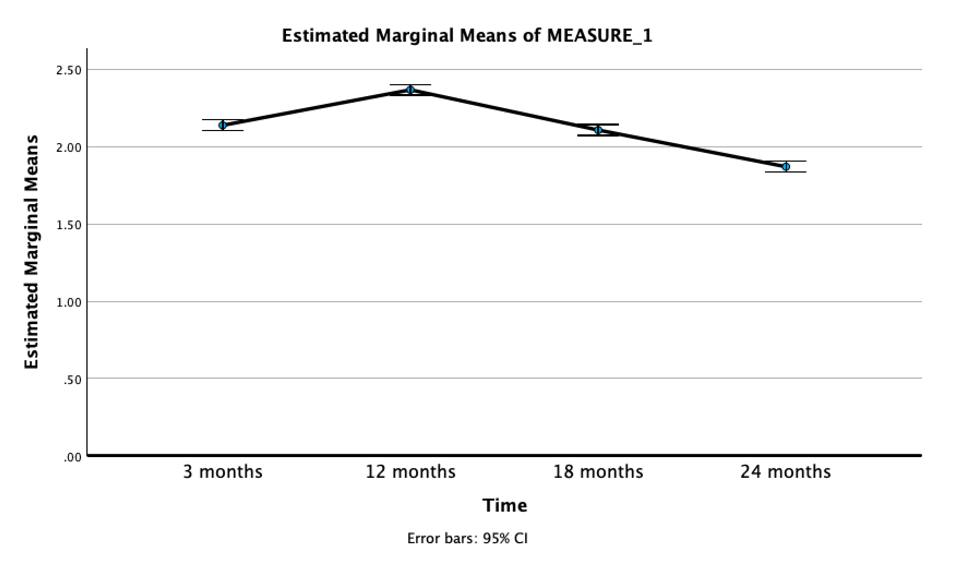
**

**Figure S6:** Perceived reliability of information from mainstream media

**
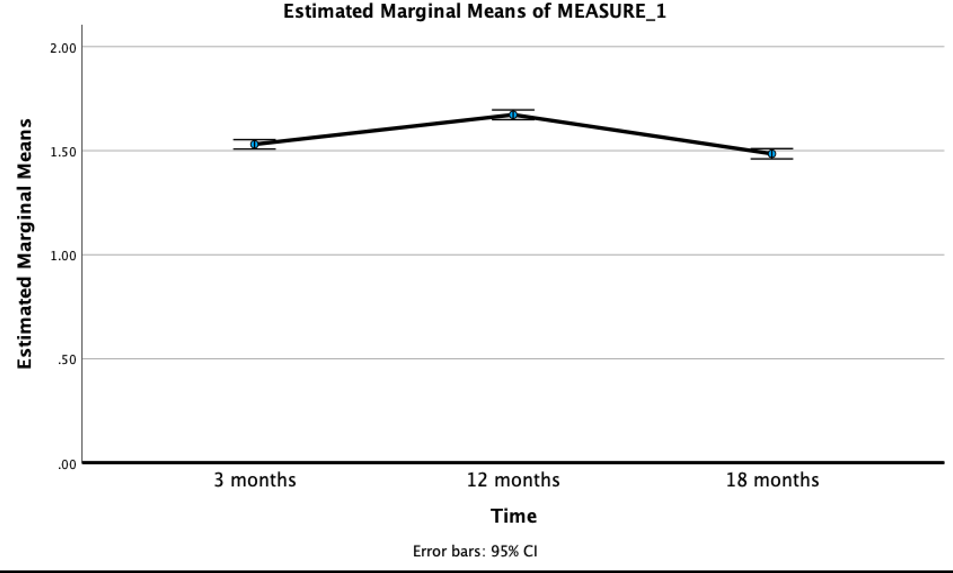
**

**Figure S7:** Perceived reliability of information from social media (participants living in Wales)

**
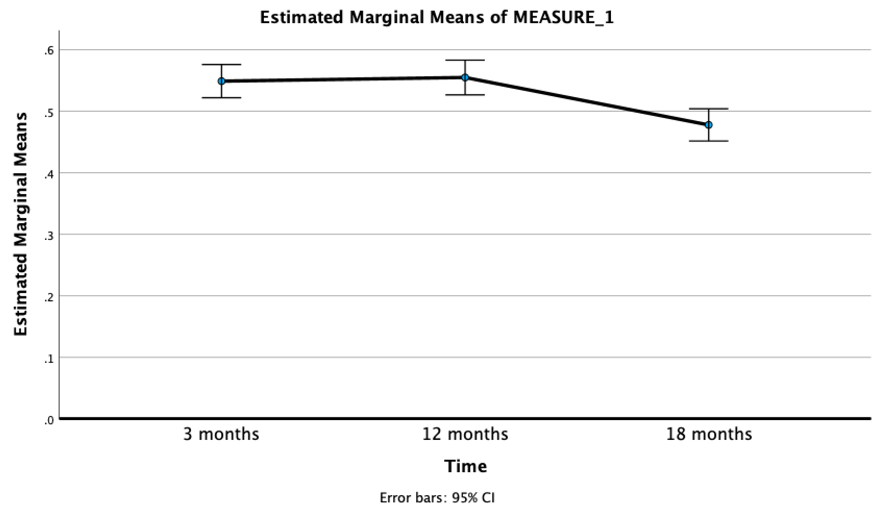
**

**Figure S8:** Perceived reliability of information from UK Government (participants living in Wales with data at all three time points)

**
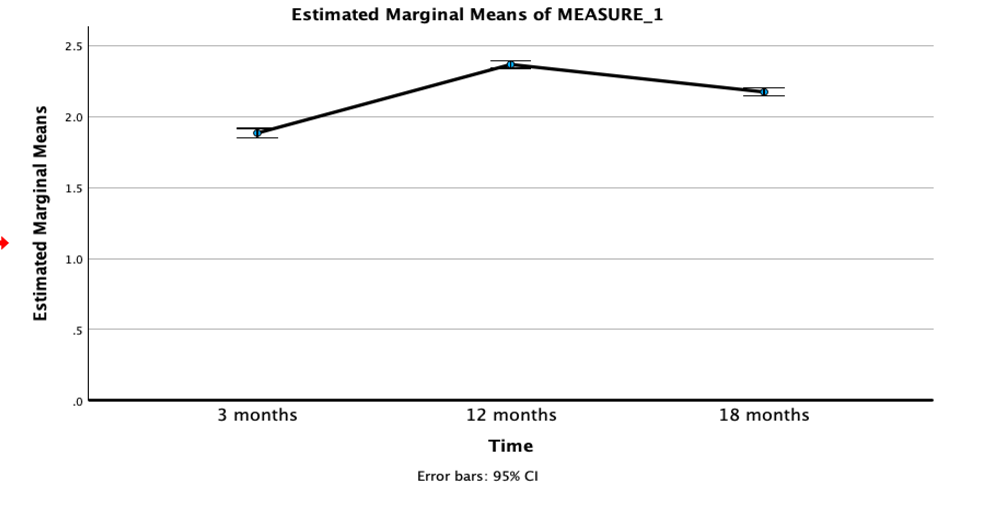
**

**Figure S9:** Perceived reliability of information from Welsh Government (participants living in Wales)


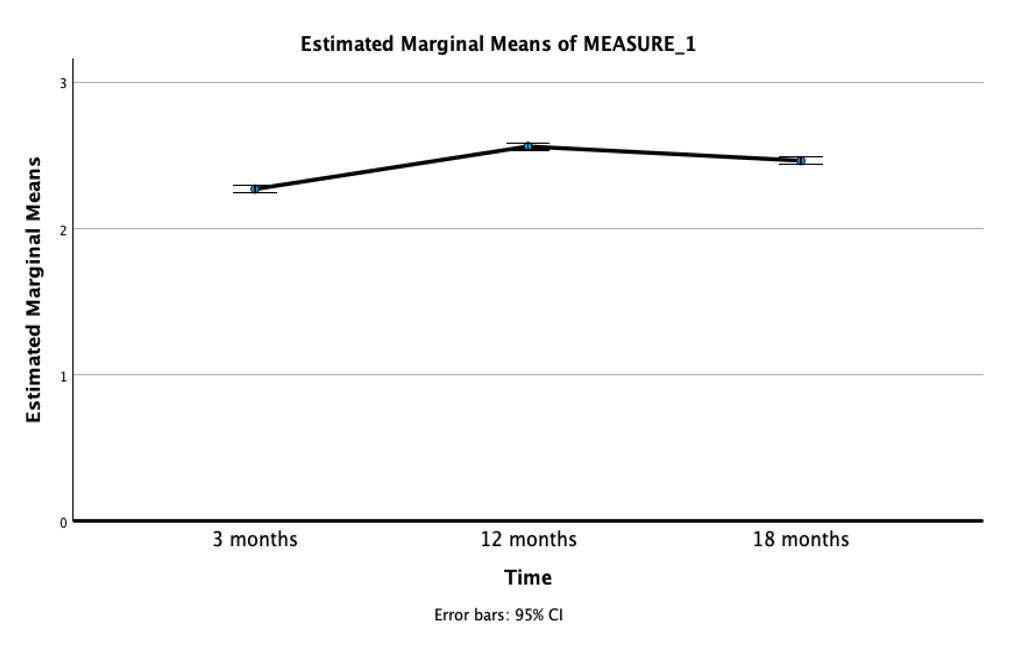

Supplement: Supplementary file 3 — File S3. [file BJHP-30-0-s004.docx]
