# Supplementary material for: Are you afraid of COVID‐19? Motivation and engagement in infection–prevention behaviour in a UK community cohort during the first 2 years of the COVID‐19 pandemic
Source: Br J Health Psychol. 2025 Nov 7;30(4):e70034. doi: 10.1111/bjhp.70034 (PMC12593319; doi:10.1111/bjhp.70034)
Supplement: Supplementary file 4 — File S4. [file BJHP-30-0-s001.docx]

# **Supplementary File 4:** Beta values for variables when trust in media and government were added to the multi-variable models at three, 12- and 18- months (data from Wales only)

| **Variable** | **3-months** | |  | **12-months** | |  | **18-months** | |  |
| --- | --- | --- | --- | --- | --- | --- | --- | --- | --- |
|  | **Beta** | **SE** | **95% CI** | **Beta** | **SE** | **95% CI** | **Beta** | **SE** | **95% CI** |
| **Perceived risk** |  |  |  |  |  |  |  |  |  |
| Susceptibility | 0.417 | 0.062 | **0.224, 0.610** | 0.403 | 0.137 | **0.133, 0.673** | 0.220 | 0.168 | -0.108, 0.549 |
| Fear of COVID-19 | 0.453 | 0.285 | **0.404, 0.502** | 0.400 | 0.033 | **0.336, 0.464** | 0.923 | 0.045 | **0.835, 1.010** |
| **Perceived behavioural control** |  |  |  |  |  |  |  |  |  |
| Protecting self | 0.267 | 0.084 | **0.103, 0.431** | 0.432 | 0.116 | **0.204, 0.660** | 0.722 | 0.149 | **0.430, 1.014** |
| Reducing spread | 0.317 | 0.067 | **0.186, 0.447** | 0.288 | 0.091 | **0.111, 0.466** | 0.586 | 0.121 | **0.348, 0.824** |
| **Health and well-being** |  |  |  |  |  |  |  |  |  |
| Subjective general health | 0.158 | 0.065 | **0.030, 0.286** | -0.160 | 0.087 | -0.330, 0.011 | -0.473 | 0.115 | **-0.698, -0.247** |
| Psychological distress (PHQ-4) | -0.062 | 0.024 | **-0.109, -0.-15** | -0.071 | 0.033 | **-0.136, -0.006** | 0.057 | 0.043 | -0.028, 0.142 |
| Believe they have had COVID-19 in last 6 months | -0.475 | 0.324 | -1.110, 0.159 | -0.891 | 0.380 | **-1.636, -0.146** | 1.286 | 0.680 | -0.049, 2.619 |
| Received one or more COVID-19 vaccinations | N/A | N/A | N/A | 0.848 | 0.273 | **0.313, 1.383** | 3.329 | 0.787 | **1.786, 4.872** |
| **Demographics** |  |  |  |  |  |  |  |  |  |
| Age category | 0.217 | 0.051 | **0.117, 0.317** | 0.249 | 0.081 | **0.091, 0.407** | 0.346 | 0.098 | **0.154, 0.537** |
| College educated | 0.314 | 0.156 | **0.008, 0.619** | 0.045 | 0.195 | -0.337, 0.428 | 0.247 | 0.270 | -0.282, 0.775 |
| Male/female | 0.657 | 0.137 | **0.388, 0.926** | 0.381 | 0.176 | **0.035, 0.726** | 0.782 | 0.235 | **0.322, 1.243** |
| **Perceived reliability of media and government information** |  |  |  |  |  |  |  |  |  |
| Mainstream media | -0.034 | 0.098 | -0.226, 0.159 | -0.177 | 0.128 | -0.429, 0.074 | -0.285 | 0.168 | -0.614, 0.045 |
| Social media | -0.269 | 0.089 | **-0.444, -0.094** | -0.296 | 0.114 | **-0.519, -0.074** | -0.454 | 0.166 | **-0.779, -0.129** |
| UK Government | -0.110 | 0.073 | -0.254, 0.034 | 0.372 | 0.121 | **0.134, 0.609** | -0.109 | 0.156 | -0.414, 0.196 |
| Welsh Government | 0.654 | 0.090 | **0.478, 0.830** | 0.832 | 0.144 | **0.549, 1.114** | 0.772 | 0.185 | **0.409, 1.136** |

*NB: Statistically significant associations (p<0.05) highlighted in bold*
